# A tribute to John Q. Trojanowski (1946–2022), neuropathologist extraordinaire

**DOI:** 10.1111/bpa.13066

**Published:** 2022-03-21

**Authors:** Michel Goedert, Maria Grazia Spillantini

**Affiliations:** ^1^ MRC Laboratory of Molecular Biology Cambridge UK; ^2^ Department of Clinical Neurosciences University of Cambridge Cambridge UK

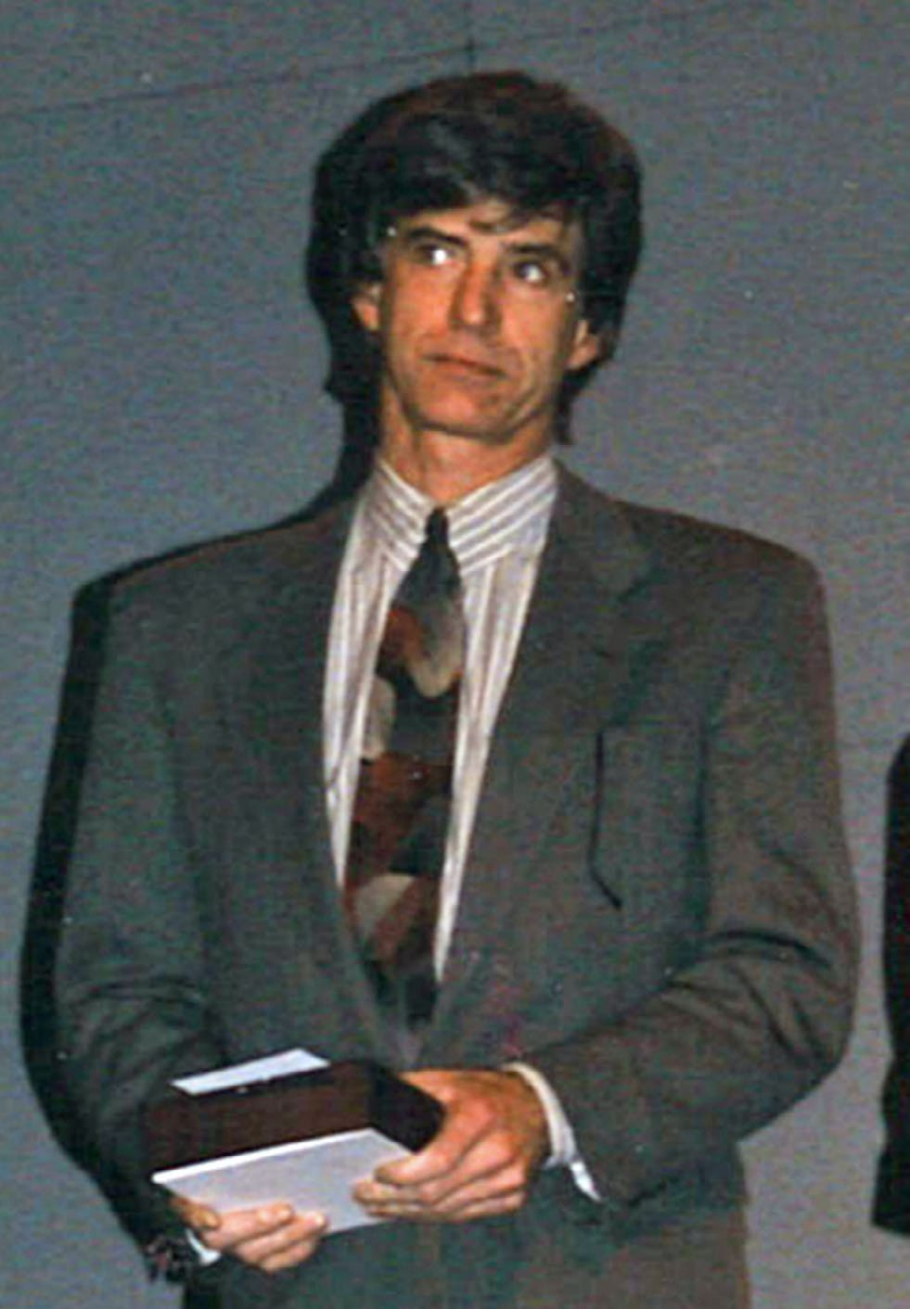

John Trojanowski, who died on February 8, was a giant in the field of neuropathology, at the forefront of research on tauopathies, synucleinopathies and TDP‐43 proteinopathies. It is less well known that he also made substantial contributions to the study of tumours of the nervous system. However, largely because of our own limitations, this article mentions only John's work on neurodegenerative diseases.

Abundant brain cell inclusions characterise most age‐related human neurodegenerative diseases. They were first identified by light microscopy following the use of newly developed silver staining techniques at the turn of the 20th century. From the 1960s onwards, electron microscopic examination showed that inclusions contain abnormal amyloid filaments. Over the past 40 years, the major components of these filaments have been identified, and their formation has been shown to be linked to the aetiology and pathogenesis of neurodegenerative diseases.

This work, to which John was a major contributor, took the study of these diseases from a descriptive to a more mechanistic level. As a result, we now speak of tauopathies, synucleinopathies, TDP‐43 proteinopathies and others. Co‐pathologies have also come to the fore. Thus, inclusions of α‐synuclein and TDP‐43 are commonly found in Alzheimer's disease (AD), together with abundant tau and Aβ inclusions.

The scientific contributions of John Trojanowski are inextricably linked with those of his partner in life, Virginia Lee. John's clinical and neuropathological expertise, together with Virginia's immunological, as well as cell and molecular biological acumen, were an ideal package.

AD, the most common neurodegenerative disease, is defined by the presence of abundant extracellular deposits and intraneuronal inclusions (plaques and tangles). In the mid‐1980s, Aβ was identified as the major plaque component. Tangles are made of paired helical and some straight filaments. Tau protein was identified as an integral component of these filaments in the mid‐late 1980s [1]. However, the insolubility of filaments isolated from tangle fragments made it difficult to exclude the presence of proteins other than tau. John and colleagues, who came to tau through work on neurofilaments, used a method for extracting more soluble filaments, to show that they were only made of tau [2]. The latter is now known to be the most commonly misfolded protein in human neurodegenerative diseases. In recent years, John and colleagues have also made important contributions to the expanding field of the prion‐like behaviour of assembled tau. Work on the enhancement of seeded tau aggregation by Aβ plaques has suggested a novel way for looking at the important, but unresolved, issue of the connection between Aβ and tau [3].

In the early 1990s, we began to collaborate with John and Virginia and have been friends ever since. John often stressed that our friendship transcended the fact that we were also scientific competitors. In 1997, we reported our most influential collaborative work, which showed the presence of α‐synuclein in the Lewy pathology of Parkinson's disease (PD) and dementia with Lewy bodies [4]. PD is the second most common neurodegenerative disease. The following year, multiple system atrophy (MSA) was shown to be the third major synucleinopathy. This work followed closely on the heels of the demonstration that dominantly inherited mutation A53T in *SNCA*, the α‐synuclein gene, causes PD.

It reinforced the paradigm that rare cases of many neurodegenerative diseases are caused by mostly dominantly inherited mutations in the genes that encode the major components of the inclusions present in all cases of disease. Thus, fewer than 1% of cases of PD are caused by mutations in *SNCA*, but at autopsy over 95% of cases diagnosed as PD (including these inherited cases) have abundant α‐synuclein inclusions in specific brain regions. This paradigm had previously been established for *PRNP*, the prion protein gene, and prion diseases, as well as for *APP*, the amyloid precursor protein gene, and AD. In 1998, the same was shown for tau, when mutations in *MAPT*, the tau gene, were found to cause inherited forms of frontotemporal dementia and Parkinsonism (FTDP‐17T) with abundant tau inclusions. John co‐authored one of three papers published in June of that year [5].

Identification of rare mutations in *SNCA* and *MAPT* in inherited cases of disease was conceptually important because it proved that dysfunction of α‐synuclein and tau is sufficient to cause neurodegeneration. At a more practical level, these findings made it possible to produce transgenic mouse lines that develop abundant filamentous inclusions, be they made of tau or α‐synuclein. John and Virginia were at the forefront of this undertaking [6, 7, 8]. Rather than being limited to investigate end‐stage human diseases, this work opened the way to more mechanistic studies. Mouse lines were also important for experimental work on the propagation of tau and α‐synuclein inclusions in brain [9, 10]. John and Virginia provided some of the best evidence in favour of the view that different conformers of assembled α‐synuclein underlie Lewy body diseases and MSA [11].

These findings left open the question of what proteins the ubiquitinated inclusions of amyotrophic lateral sclerosis (ALS) and some forms of frontotemporal lobar degeneration (FTLD) are made. John and Virginia worked on this difficult problem for many years, with a combination of immunology and protein chemistry eventually resulting in their demonstration that TAR DNA‐binding protein‐43 (TDP‐43) is the major component of these inclusions [12]. TDP‐43 had not previously been linked to neurodegenerative disease. Inclusions are found in around 97% of cases of ALS and 50% of cases of FTLD. This discovery opened a new chapter in the study of neurodegenerative diseases. It confirmed that ALS and the most common form of FTLD belong to a clinicopathological spectrum and indicated that RNA mismetabolism may play an important role. This concept was subsequently extended through the demonstration that dysfunction of other RNA‐binding proteins, such as fused in sarcoma (FUS), Ewing sarcoma RNA‐binding protein‐1 (EWSR1) and TATA‐binding protein‐associated factor‐15 (TAF15) is central to some cases of ALS and FTLD.

Mutations in *TARDBP*, the TDP‐43 gene, cause a small number of inherited cases of FTLD with abundant TDP‐43 inclusions, similar to what has been observed for *PRNP* in prion diseases, *APP* in AD, *SNCA* in PD and *MAPT* in FTDP‐17T. Besides sporadic cases of FTLD, TDP‐43 inclusions are also found in inherited cases caused by mutations in *GRN* (granulin gene), *VCP* (valosin‐containing protein gene) and *C9orf72* (chromosome 9 open reading frame 72). Like tau and α‐synuclein, assembled TDP‐43 from brain can induce the formation and spreading of inclusions [13]. Subtyping based on the appearance and distribution of inclusions has been particularly influential in the context of FTLD‐TDP [14]. Moreover, it is now also recognized that a TDP‐43 proteinopathy is commonly found in subjects who are over 80 years old. This entity has been named LATE (limbic‐predominant age‐related TDP‐43 encephalopathy) by John and colleagues [15].

John had an attractive and whirlwind personality. He was a warm, cultured and urbane individual, with a well‐developed sense of humour. We will miss him dearly. It is difficult to believe that we shall never again see John step up to the microphone and say ‘John Trojanowski, University of Pennsylvania’, before asking a pertinent question. To remember what John was like, watch the YouTube video film of his ALS Ice Bucket Challenge from August 2014: (https://www.youtube.com/watch?v=fdf12InZ‐K8&feature=youtu.be).
